# Proteomic Analysis of the *Schistosoma mansoni* Miracidium

**DOI:** 10.1371/journal.pone.0147247

**Published:** 2016-01-22

**Authors:** Tianfang Wang, Min Zhao, Bronwyn A. Rotgans, April Strong, Di Liang, Guoying Ni, Yanin Limpanont, Pongrama Ramasoota, Donald P. McManus, Scott F. Cummins

**Affiliations:** 1 Genecology Research Centre, University of the Sunshine Coast, Maroochydore DC, Queensland, 4558, Australia; 2 School of Medical Science, Griffith Health Institute, Griffith University, Gold Coast, Queensland, 4222, Australia; 3 Faculty of Tropical Medicine, Mahidol University, Bangkok, 10400, Thailand; 4 QIMR Berghofer Medical Research Institute, Brisbane, Queensland, 4006, Australia; University of Rochester, UNITED STATES

## Abstract

Despite extensive control efforts, schistosomiasis continues to be a major public health problem in developing nations in the tropics and sub-tropics. The miracidium, along with the cercaria, both of which are water-borne and free-living, are the only two stages in the life-cycle of *Schistosoma mansoni* which are involved in host invasion. Miracidia penetrate intermediate host snails and develop into sporocysts, which lead to cercariae that can infect humans. Infection of the snail host by the miracidium represents an ideal point at which to interrupt the parasite’s life-cycle. This research focuses on an analysis of the miracidium proteome, including those proteins that are secreted. We have identified a repertoire of proteins in the *S*. *mansoni* miracidium at 2 hours post-hatch, including proteases, venom allergen-like proteins, receptors and HSP70, which might play roles in snail-parasite interplay. Proteins involved in energy production and conservation were prevalent, as were proteins predicted to be associated with defence. This study also provides a strong foundation for further understanding the roles that neurohormones play in host-seeking by schistosomes, with the potential for development of novel anthelmintics that interfere with its various life-cycle stages.

## Introduction

The schistosome life cycle is complex, involving a number of different stages including larval cercariae, shed from freshwater intermediate host snails which subsequently penetrate the skin of their human or other mammalian definitive hosts. On successful invasion, cercariae develop into schistosomula and mature into adult male and female worms which pair up and migrate to the portal mesenteric system of the mammalian host, where the females lay eggs. Some of the eggs leave the host and hatch in water as free-living miracidia. The miracidia seek out intermediate host snails, thus completing the schistosome life cycle.

The publication of the genomes for *Schistosoma japonicum* and *Schistosoma mansoni* have significantly advanced our understanding of schistosome biology [1,2]. Most interest to date has been devoted to using these data to interpret the mechanisms of interplay between schistosomes and their mammalian hosts, and the development of new control interventions including vaccines or additional therapies to praziquantel [3,4,5,6]. However, insufficiency in controlling the disease by chemotherapy alone has been increasingly noticed [7]; in addition, the possibility of the emergence of drug resistant parasites has been raised [8,9]. A feasible alternative is to decipher the process in which miracidia invade their intermediate snail hosts to develop new strategies to prevent attraction and/or invasion of snails, thereby preventing transmission. With this goal in mind, an international consortium was initiated to sequence the genome of *Biomphalaria glabrata*, the intermediate host of *S*. *mansoni* [10]. As such, *S*. *mansoni miracidia* and *B*. *glabrata* represent a highly tractable model for analysis of the snail host-parasite relationship. The interaction between *B*. *glabrata* and water-borne free-living schistosome miracidia is multifaceted. From the perspective of snail, studies have suggested that macromolecular glycoconjugates released by snails seem to be miracidium attractants [11,12]. Following invasion, *B*. *glabrata* often fails to activate an appropriate immune response against *S*. *mansoni*. The parasite consequently develops successfully in these susceptible snails, resulting in the release of infective cercariae several weeks post-exposure.

The proteomic analysis of different subset proteins of Schistosome relevant to the infection process of snail, including immature egg [13,14], egg secreted proteins/secretome [15,16], egg contents [13,17], excretory-secretory proteins of miracidia *in vitro* [18] and sporocysts [19,20], has been advancing the progress of unveiling the snail-parasite interaction mechanism. Mucins of *S*. *mansoni* were recognised as key factors effecting the interaction between miracidia/sporocysts and host snail [17]. Chaperones, such as Hsp70, 60 and 90, were identified as the most abundant protein family in egg development, while defence proteins were enriched in the hatch fluid [13]. Cytoskeletal proteins were with similar expression levels in the immature eggs and mature eggs contents [13]. The synthetic activity of mature eggs was found to depend on the secreted proteins, which included two proteases [15]; a later study identified 188 proteins from the secretome of eggs, playing roles in redox balance, development, protein folding and molecular chaperoning, and so forth [16]. At the *in vitro* miracidium-to-sporocyst stage, 127 identified larval transformation proteins mainly composed of proteases/protease inhibitors, small HSPs, redox/antioxidant enzymes, ion-binding proteins, and venom allergen-like (VAL) proteins [20].

The characterisation of the proteome of the *S*. *mansoni* miracidium has not been reported, possibly due to the difficulty in purifying eggs of *S*. *mansoni* out of the debris of mammalian organs, which results in low quality proteomic data for miracidia. The present study utilised a protocol to obtain *S*. *mansoni* eggs with minimal mammalian host contamination, and characterised the proteome of miracidium at the stage of 2hrs post-hatch using high-throughput LC-MS/MS screening. At about 2hrs post-hatch the miracidium has completed development, initiates host finding and meanwhile starts to die [21,22]. Secreted proteins and chemosensory receptors are at the forefront of snail-miracidia interaction, and were therefore of particular interest for investigation. This information furthers our understanding of the complete schistosome proteome, and provides the foundation for further investigations into the molecular basis of schistosome modulation of snail-host immunity and host finding. This increases the possibility of identifying those factors that promote parasite resistance in *B*. *glabrata*, and may aid the development of new approaches to control snail infection, so that schistosomiasis can be tackled from a new angle.

## Materials and Methods

### Ethics statement

The conduct and procedures involving animal experimentation were approved by the Animal Ethics Committee of the QIMR Berghofer Medical Research Institute (project number P242). This study was performed in accordance with the recommendations in the Guide for the Care and Use of Laboratory Animals of the National Institutes of Health.

### Collection of *Schistosoma mansoni* miracidia and protein extraction

We used ARC Swiss, outbred mice which were from the Animal Resource Centre in Western Australia. The Puerto Rican strain of *S*. *mansoni* is maintained, under permit from the Australian Department Agriculture, Fisheries and Forestry Biosecurity (DAFF), in ARC Swiss mice and *Biomphalaria glabrata* snails at QIMR-Berghofer Medical Research Institute (QIMR-B) from stock originating from the National Institute of Allergy and Infectious Diseases Schistosomiasis Resource Centre, Biomedical Research Institute (Rockville, Maryland, USA).

#### Isolation of Schistosome eggs

Mice were euthanized with CO_2_ gas and their livers were perfused with chilled PBS. Eggs of *S*. *mansoni* were collected during perfusion of mice. Four infected mouse livers were sliced with scalpel blades and blended to a smooth consistency in 50 mL phosphate buffered saline (PBS). *S*. *mansoni* eggs formed a very firm pellet at the bottom of the tubes, which was incubated with collagenase B (20 mg) and 110 μL Pen/strep (or 10 μg penicillin and 20 μg streptomycin) with at 37^°^C in a shaker overnight [23].

The pellet was washed twice using PBS with centrifugation at 400 x g for 5 min, in a final volume of 25 mL PBS. This mixture was filtered successively through 250 μm and 150 μm sieves and subjected to centrifugation at 400 x g for 5 min, and the pellet resuspended in 10 mL PBS. This mixture was placed onto a Percoll column (8 mL Percoll + 32 mL of 0.25 M sucrose in 50 mL tube), which was centrifuged at 800 x g for 10 min. Liver cells were removed from the top of the Percoll column with a Pasture pipette.

The egg pellet was retained within 25 mL PBS containing 1 mM EDTA, 1 mM EGTA, centrifuged at 30 x g for 3 min, then the discarded supernatant was mixed with 10 mL of the same buffer and centrifuged at 30 x g for 3 min, and the procedure was repeated. The egg pellet was resuspended in 5 mL PBS and applied on to a second Percoll column (2.5 mL Percoll + 7.5 mL 0.25 M sucrose in a 15 mL tube), followed by centrifugation at 800 g for 10 min. The supernatant was removed and the egg pellet washed with 10 mL PBS and then centrifuged at 30 x g for 3 min. The washing with PBS was repeated three times.

#### Isolation of miracidia

Eggs were transferred into a 200 mL hatching measuring cylinder wrapped completely in light-blocking black tape with the exclusion of the top 4 cm from the lip, thereby producing a light-gradient. The hatching cylinder was topped with conditioned water (Milli-Q water stored with calcium carbonate chips until pH ≈ 7) until above the tape-covered area ~1.5 cm and exposed to bright light at 27^°^C. Eggs were incubated for 2 h post-hatch before collection of the top 10 mL of miracidium-containing water (MCW) above the light-blocking region. Hatched miracidia were collected by centrifugation at 8,000 x g for 1 min at 4^°^C, and were then washed twice with water.

#### Protein extraction from miracidia

Miracidia pellets at 2h post-hatch were used for protein extraction using urea/thiourea buffer [7 M urea, 2 M thiourea, and 4% CHAPS, in 30 mM Tris-HCl (pH 8.5)] with a protease inhibitor cocktail (GE Healthcare Life Sciences), and homogenised. The lysate was allowed to settle at room temperature for 10 min; subsequently, the sample lysate was centrifuged at 15,000 x g for 15 min. The supernatant was collected for sodium dodecyl sulphate-polyacrylamide gel electrophoresis (SDS-PAGE).

### SDS-PAGE and trypsin digestion

Lysate proteins were size fractionated by 1D SDS-PAGE with a 4–12% polyacrylamide gradient gel (Amersham ECL Gel, GE Healthcare Life Sciences) according to the manufacturer’s instructions. The gel was stained with Coomassie Brilliant Blue R250 (Sigma-Aldrich). Two lanes of biological replicates (about 150 miracidia each) containing protein were excised (equally cut into 51 pieces in each lane) and processed for LC-MS/MS. Briefly, proteins within each gel piece were subjected to reduction (10 mM dithiothreitol, DTT, 45 min at 56^°^C) and alkylation (iodoacetamide, IAA, 30 min, RT, in the dark) followed by Sequencing Grade Modified Trypsin (Promega) for 16 h at 37^°^C. Peptides were then extracted from the gel pieces as described [24], desalted and concentrated by Ziptip C18 (Millipore).

### NanoHPLC-ESI-TripleTOF Analysis

Purified extracts (after Ziptip) were analysed by LC-MS/MS on a Shimadzu Prominance Nano HPLC (Japan) coupled to a Triple Tof 5600 mass spectrometer (ABSCIEX, Canada) equipped with a nano electrospray ion source. The protocol has been detailed elsewhere [25]. Briefly, approximately 6 μL of each extract was injected and de-salted on the trap column before entering a nano HPLC column (Agilent Technologies, Australia) for mass spectrometry analysis. The mass spectrometer acquired 500 ms full scan TOF-MS data followed by 20 by 50 ms full scan product ion data. Full scan TOFMS data was acquired over the mass range 350–1800 and for product ion MS/MS 100–1800. Ions observed in the TOF-MS scan exceeding a threshold of 100 counts and a charge state of +2 to +5 were set to trigger the acquisition of product ion. The data were acquired and processed using Analyst TF 1.5.1 software (ABSCIEX, Canada).

### Protein identification

The *S*. *mansoni* protein sequence database (ASM23792v2.27) was downloaded from the *Schistosoma mansoni* database of EnsemblMetazoa website (http://metazoa.ensembl.org/Schistosoma_mansoni/Info/Index), with a total number of 11,774 sequences. To improve protein identification and coverage, four search engines were comparatively used to analyse the mass spectrometric data, including PEAKS (Bioinformatics Solutions Inc., Waterloo, ON, Canada, version 7.0), X! Tandem [26] (version 2009.04.01), MS Amanda [27] (version 1.0.0.5242) and OMSSA [28] (version 2.1.7). A composite target−decoy database was built with the forward and reverse sequences for calculating the false discovery rate.

All searches used the following search parameters: precursor ion mass tolerance, 0.1 Da; fragment ion mass tolerance, 0.1 Da; fully tryptic enzyme specificity; two missed cleavage; the number of unique peptide ≥1; monoisotopic precursor mass (PEAKS and OMSSA); monoisotopic fragment ion mass (OMSSA); a fixed modification of cysteine carbamidomethylation; and variable modifications included methionine oxidation, conversion of glutamine and glutamic acid to pyroglutamic acid, and deamidation of asparagine. For PEAKS, *de novo* sequencing, database search and characterising unspecific post-translational modifications (PTMs) were used to maximise the identifications; false discovery rate (FDR) was set to ≤ 1%, and the individual peptide ion score [-10*Log(p)] was calculated accordingly, where p is the probability that the observed match is a random event. An additional parameter unique to OMSSA was used that requires that one of a variable number of the most intense fragment ions match those of the theoretical peptide; in this case the parameter was set to 5. For other three engines except PEAKS, the confidence was set to greater than 95%.

### Gene ontology, KEGG pathway and prediction of secreted proteins

Identified proteins were firstly subject to BLASTp and tBLASTn using non-redundant protein sequences and nucleotide collection of NCBI, respectively. Then, BLAST results were combined and imported to BLAST2GO[29] (version 3.1), to perform gene ontology (GO) and KEGG pathway analysis. Fisher’s exact test was carried out to evaluate the enrichment of GO terms in miracidium proteome (test dataset) against the annotation of all *S*. *mansoni* proteins (reference dataset). The significant GO terms with *p*<0.01 were considered as over-represented, and FDRs were calculated from p-values using the Benjamini-Hochberg procedure [30].

Protein N-terminal signal sequences were predicted using the SignalP 4.1 [31] and Predisi [32], with the transmembrane domains predicted by TMHMM [33]. For SignalP predictions, positive identifications were made when both neural network and hidden Markov model algorithms gave coincident estimations; D-cutoff values were set to 0.34 (to increase sensitivity) for both SignalP-noTM and TM networks. Herein, a protein was designated as secreted, only when it met the criteria of both SignalP and Predisi, and did not have a transmembrane domain predicted by TMHMM.

### Identification of neuropeptide genes

To identify target sequences, previously identified platyhelminth (flatworm) neuropeptide genes were used as query (BLASTn and BLASTp) against the *S*. *mansoni* genome that was imported into the CLC genomics workbench (version 7.0.3). Six target neuropeptide genes were compared to known platyhelminth neuropeptides using multi-sequence alignments constructed in the Molecular Evolutionary Genetics Analysis (MEGA) software version 6.0.5 [34]. Similar homology comparisons were utilized through the NCBI database (http://blast.ncbi.nlm.nih.gov/Blast.cgi) using BLASTp and BLASTx searches. Sequence presentation and shading of multiple sequence alignments was performed using the LaTEX TEXshade package [35]. Relative gene expression of target neuropeptide genes between cercariae, 3S, 24S and adult schistosomes (as relative normalized reads) were obtained from SchistoDB [36] (http://SchistoDB.net) for *S*. *mansoni*.

To identify neuropeptide gene expression in miracidia, total RNAs were prepared from the 2 h post-hatch larvae using TRIzol reagent (Invitrogen), following the manufacturer’s protocol. Purity and quantity was measured using a UV spectrophotometer (NanoDrop ND-1000) at 260 and 280 nm. First-strand cDNA was generated from miracidia total RNA using random hexamer primers and the Superscript Preamplification System for First-strand Synthesis (Invitrogen). RT-PCR was performed using primers designed on CLC genomic workbench (version 7.0.3) (**Table 1**), with actin used as a positive control, inclusive of amplification without cDNA as negative control. Samples were heated at 94°C (2 min), amplified for 35 cycles (repetition of 30 s at 95°C, 30s at 50°C and 1 min at 72°C) and extended at 72°C (5 min). Samples were separated on agarose gel (2%, ethidium bromide) for visualisation.

**Table 1:** Primers used for RT-PCR of neuropeptide genes.

Gene	Sequence 5’ to 3’
**GFVRIamide**	
Forward:	AAACAGTGATGGCTTACCAA
Reverse:	TGAAACAACTGACTACCGAA
**RGMIamide**	
Forward:	CTACAACAACAATCACCCAC
Reverse:	TAAACCACTTCCTGCAAAAC
**PWamide**	
Forward:	TTTCCCACCATCATCATC
Reverse:	CTTACATTTCACCTTCAGTC
**RLamide**	
Forward:	ATCACCAACCAACATCATAG
Reverse:	AGTTCAGCAACATTATCACC
**Neuropeptide F (NPF)**	
Forward:	TCCATTTCCTTCTCTTCTCTCT
Reverse:	TCTCCTGACGTTGTTCTACT
**Actin**	
Forward:	CTCTCACTCAATCACTAACTC
Reverse:	ACATAGCTGGCACATTAAAC

## Results

### *S*. *mansoni* miracidia proteomic analysis and protein identification

**Fig 1:**
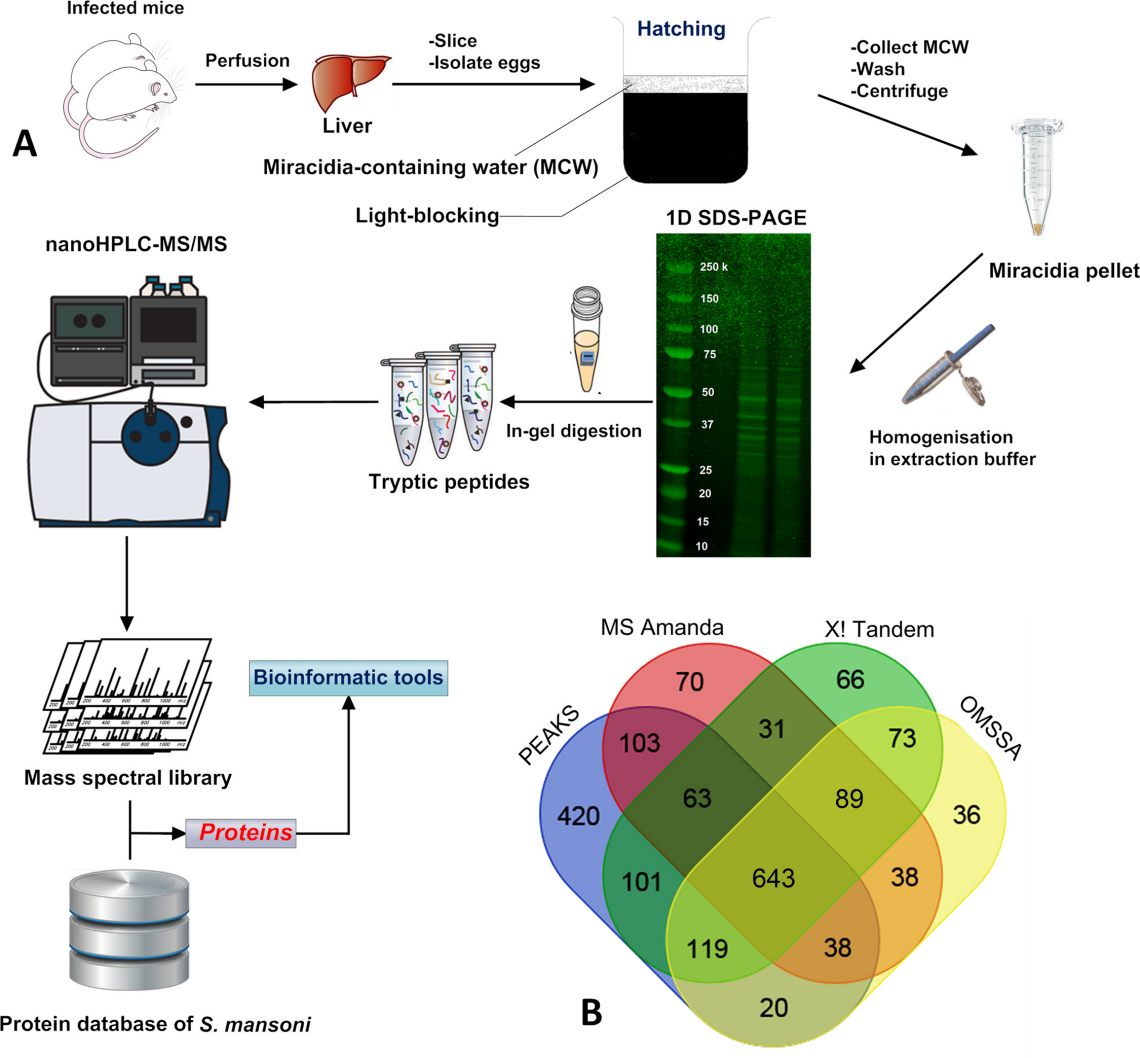
(A) Overall workflow for *S*. *mansoni* egg isolation, protein extraction and identification from miracidia. Miracidium proteins were extensively fractionated followed by identification with high-accuracy nano-LC TripleTOF MS. **(B)** Protein identification results of different search engines (see Methods for search parameter settings).

The overall experimental procedure to map and annotate miracidia proteins encoded by the *S*. *mansoni* genome is outlined in **Fig 1A**. Miracidia proteins were extensively fractionated by 1D SDS-PAGE, followed by in-gel digestion. All samples were subjected to high-accuracy mass spectrometry, and the raw data were rigorously analysed using available informatics tools. Based on the genome of *S*. *mansoni* published in 2009, it was estimated that there are at least 11,809 genes (encoding 13,197 transcripts in mixed-sex cercariae), of which certain features were described, including unusual intron size distribution and new families of micro-exon genes that undergo frequent alternative splicing [1]. The protein database in our study contained 11,774 different sequences, from which we have identified a total of 1,910 *S*. *mansoni* proteins integrating the results of four search engines with high confidence (one or more unique peptides with an FDR less than 1%) in miracidia (**S1 Table**). The numbers of proteins identified by four search engines are compared in **Fig 1B**. Overall, there are 643 proteins mutually identified by all engines; PEAKS have 420 sequences uniquely identified, compared to 70, 66 and 36 by MS Amanda, X! Tandem and OMSSA. There are a number of proteins are crossed identified among different engines. This offers the highest number of proteins identified from miracidia, providing ~16.2% of the total proteins predicted from the *S*. *mansoni* genome (11,774 entries, *Jul*. 2015). As it has been reported that around 6000–7000 genes are expressed in each *S*. *mansoni* stage [37], then the number of proteins we have identified may cover approximately 32–45% of those expressed proteins.

### Gene ontology analysis

**Fig 2:**
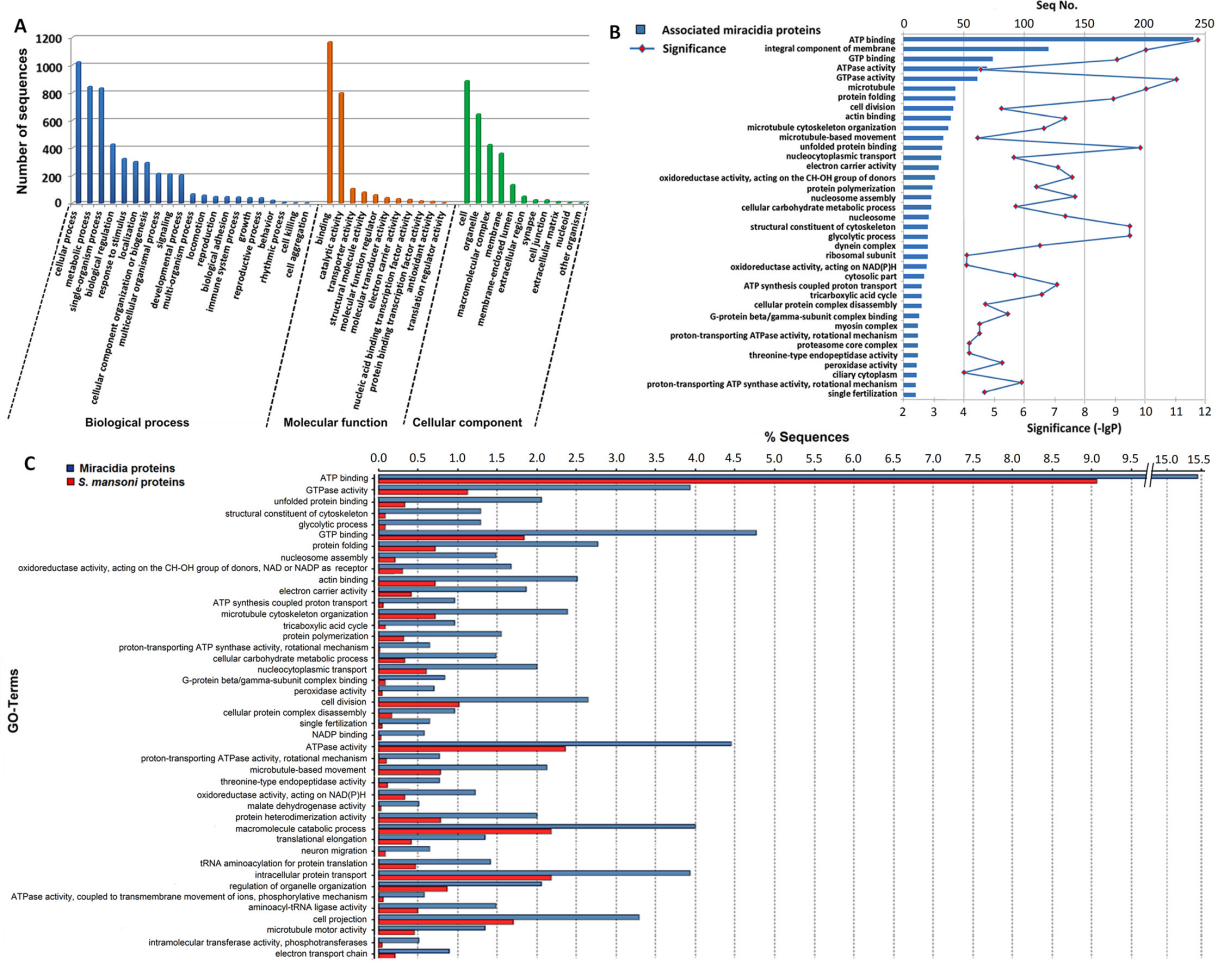
Gene ontology annotation of miracidia proteins. The bar chart (A) describes the GO distribution of GO term on level 2 from the proteome. When one protein was known to be localised in more than one cellular compartment, all of the localisations were counted non-exclusively. (B) All identified miracidia proteins were compared with the whole protein database of *S*. *mansoni*. Significantly over-represented GO terms (*p*<0.01) are shown. The number of proteins and their levels of significance are also shown. (C) GO terms of biological process and molecular function over-represented in miracidia compared to the whole *S*. *mansoni* proteins, in terms of the ratio of protein numbers.

The distributions of the GO term (the second level) of proteins identified for the three categories of biological process, molecular function and cellular component are shown in **Fig 2A**, while the distributions of the GO term at all levels of the LC-MS/MS identified proteins for the same three categories are recorded in **S2 Table**. There is also a large category comprised of proteins without any GO term prediction (15.4%, **Table 2**), including 41 novel proteins (no BLAST result meeting the eValue cutoff). Cellular and metabolic processes are the two most highly represented biological process terms in the *S*. *mansoni* miracidium proteome, each containing over 800 proteins. There was also a high presence of miracidial proteins implicated for roles in basic regulation and responses specifically relevant to single organisms, as well as for responding to stimuli, probably a requirement for movement post-hatch, seeking out a host snail and host penetration. Approximately 198 proteins are associated with signalling and cell-cell signalling, respectively (**S2 Table**); these are characteristic components of those systems requiring multicellular processes and communication.

**Table 2:** Categories of proteins identified from *S*. *mansoni* miracidia, annotations with/without GO terms, predicted as secreted proteins and receptors

Sequence number	Miracidia
Identified, in total	1910
With GO term(s)	1616
Without GO term(s)	250
Secreted	75
Receptors	37

GO of proteins related to molecular function show that the majority of proteins identified in this study are involved in binding (61.3%) and catalytic activity (42.0%) (**Fig 2A**). Within the binding group, organic cyclic compound binding (34.3%), ion binding (32.4%), and protein binding (29.4%)and are the top three categories. Meanwhile, within the catalytic activity group, hydrolase activity (20.3%) and transferase activity (13.3%) are the two categories containing largest number of sequences (**S2 Table**). Most hydrolase activity-type and transferase activity-type proteins participate in the catalysis of glycosylation hydrolysis, as well as in transferring phosphorus-containing and glycosyl groups. Miracidium proteins with GO under the cellular component category included those with cell (46.5%), organelle (33.9%), macromolecular complex (22.4%), membrane (19.1%), membrane-enclosed lumen (7.2%), extracellular region (2.8%), cell junction (1.4%), synapse (1.4%) and extracellular matrix (0.1%) activity. In addition, there are 294 proteins that do not match to any GO category (**S2 Table**).

The fisher’s exact test of the GO is displayed in **Fig 2B**, with enriched terms listed in **S3 Table**. It can be seen that the processes of carbohydrate breakdown (*p* = 3.18E-10) are significant in miracidia 2h post-hatch. Specific events such as protein folding (*p* = 1.10E-9) and nucleosome assembly (*p* = 2.07E-8) are also enriched. These processes are important for energy production, protein functionalisation and DNA replication. We also found significant overrepresentation of proteins in ATP synthesis coupled proton transport. In terms of molecular function, statistical analysis shows a very significant enrichment of ATP/GTP binding and ATPase/GTPase activity. In comparison to the pooled *S*. *mansoni* database, we found a high ratio of the proteins involved in cell projection, intracellular protein transport, macromolecule catabolism, microtubule-based movement and cytoskeleton organisation, cell division, nucleocytoplasmic transport and protein polymerisation (**Fig 2C**). The under-represented molecular function in miracidia included G-protein coupled receptor activity and sequence-specific DNA binding transcription factor activities (**S3 Table**).

### KEGG pathway analysis

**Fig 3:**
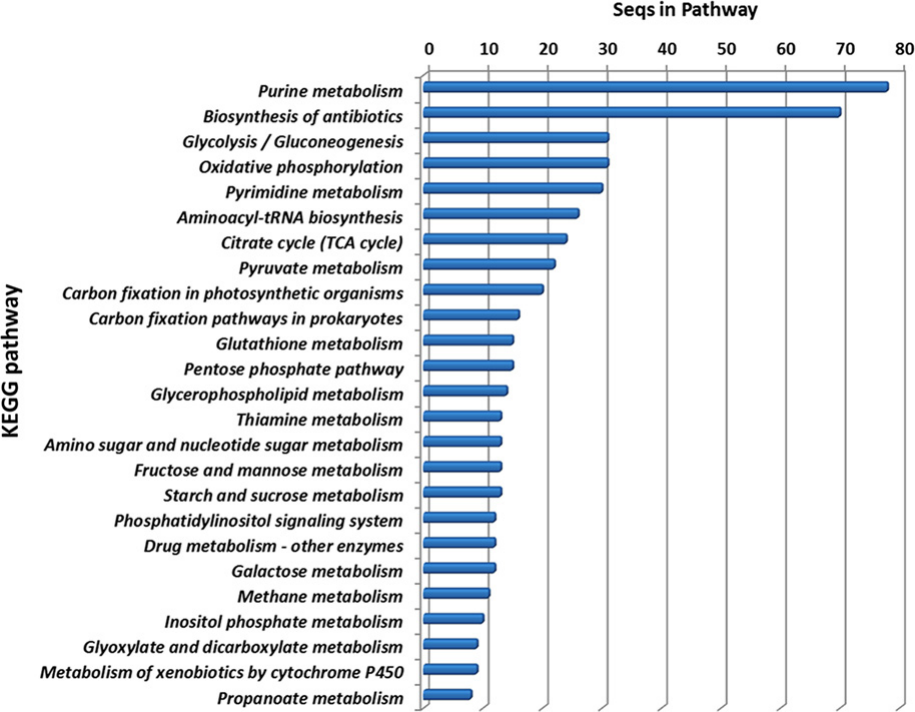
KEGG analysis of miracidium proteins showing the top 25 highly represented KEGG pathways (see S2 Table for the list of all KEGG pathways identified).

Using the KEGG pathway database, the *S*. *mansoni* miracidium proteins identified can be classified into 95 pathways (**S1 Fig** and **S2 Table**), the top 25 of which are shown in **Fig 3**. Purine metabolism is the top pathway represented, followed by the protein processing pathway of biosynthesis of antibiotics, which includes 70 sequences. There are also a significant number of proteins present that relate to pathways involved in energy procurement, e.g., 31 sequences playing roles in glycolysis/gluconeogenesis, oxidative phosphorylation and pyrimidine metabolism, respectively. Besides, a number of proteins relate to sugar metabolism, including the metabolism of fructose and mannose (13 seqs), starch and sucrose metabolism (13), amino and nucleotide sugar (13), and galactose (12) (**Fig 3**). In addition, proteins were identified that are associated with inositol phosphate metabolism and the citrate cycle (TCA cycle), which could play crucial roles in a diverse number of cellular functions, such as cell growth, apoptosis, cell migration, endocytosis, and cell differentiation [38]. The TCA cycle is the final pathway of metabolism of three nutrients—carbohydrates, lipids, and amino acids, so the presence of these pathway proteins may be attributed to the process of nutrient synthesis and storage in miracidia.

In addition, 15 proteins can be attributed to glutathione metabolism. Glutathione (GSH) works as an antioxidant molecule in plants, animals, fungi and some bacteria and archaea, preventing damage to important cellular components [39]. A similar role is likely for this group of proteins in the miracidium. So far, several glutathione metabolism relevant proteins have been considered as antischistosomal drug candidates, such as thioredoxin glutathione reductase (TGR) [40,41] and glutathione-S-transferase [42], both of which were identified in this study.

### Secreted proteins in *S*. *mansoni* miracidia

Of the 1,910 miracidium proteins identified, 75 are predicted by SignalP, Predisi and TMHMM to be secreted (**Table 3 and S2 Table**). Secreted proteins may be involved in a variety of physiological processes in the miracidium, including cell signalling and cell-cell communication. We identified various members of the venom allergen-like (VAL) protein family (**Table 4**), and VAL 2, 3, 9, 26, 27 and 28 are predicted to be secretory.

**Table 3:** List of identified secreted proteins with -10*Log(P)≥50 from *S*. *mansoni* miracidia (see S2 Table for the full list).

Sequence name	Sequence description	-10*Log(P)	#Peptides	Mass (kDa)
Smp_049550.1:pep	78 kda glucose-regulated protein	335.14	31	71.2
Smp_179250.1:pep	alpha-galactosidase, alpha-n-acetylgalactosaminidase	275.55	25	47.8
Smp_079770.1:pep	protein disulfide isomerase-associated 3 precursor	251.47	15	54.4
Smp_056760.1:pep	protein disulfide-isomerase	207.21	14	54.2
Smp_030300.4:pep	endoplasmin	183.37	20	239.5
Smp_089670.1:pep	alpha-2-macroglobulin-like protein 1	181.61	13	229.9
Smp_021730.2:pep	cytochrome c oxidase subunit Vb: cox4	171.7	9	25.1
Smp_150240.1:pep	secretory glycoprotein k5 precursor	168.2	7	31.2
Smp_098420.1:pep	hypothetical protein (*function unknown*)	159.57	8	36.5
Smp_112110.1:pep	interleukin-4-inducing protein precursor	155.09	4	15.4
Smp_154290.1:pep	glipr1-like protein 1 precursor	152.75	7	20.7
Smp_143150.1:pep	elongation factor 2	151.28	10	60.7
Smp_020340.1:pep	lysosomal alpha-glucosidase	137.23	7	101.6
Smp_032670.2:pep	egg protein c122-like	137.1	4	17.2
Smp_030370.1:pep	calreticulin	132.13	7	45.4
Smp_172110.1:pep	protein disulfide-isomerase	120.47	3	40.2
Smp_017730.1:pep	GPI anchored surface glycoprotein	117.25	6	186.5
Smp_180530.2:pep	phosphoglucomutase	114.35	3	62.7
Smp_160560.1:pep	iron dependent peroxidase	113.15	5	79.5
Smp_145300.1:pep	peptidylglycine alpha-hydroxylating monooxygenase	110.24	2	25.4
Smp_199860.1:pep	6-phosphogluconate decarboxylating	109.65	3	17.6
Smp_155890.1:pep	alkaline phosphatase	90.67	4	46.3
Smp_089640.2:pep	succinate dehydrogenase	86.78	4	32.3
Smp_159800.1:pep	hypotheticial protein (*function unknown*)	78.58	1	9.1
Smp_000260.1:pep	proactivator polypeptide	78.41	1	104.7
Smp_005710.1:pep	egg protein cp391s-like	76.57	3	41.7
Smp_056200.1:pep	isocitrate dehydrogenase	65.77	2	43.5
Smp_088720.1:pep	nadh:ubiquinone oxidoreductase complex I intermediate-associated protein-containing protein	63	3	27.4
Smp_179560.1:pep	multiple inositol polyphosphate phosphatase	54.7	2	59.2

**Table 4:** Venom allergen-like (VAL) proteins identified by from *S*. *mansoni* miracidia (also see S1 Table for more details).

Accession	-10*Log(P)/Confidence	Coverage (%)	#Peptides	Mass (kDa)	Description
Smp_120670.1:pep	153.53	25	7	16.4	VAL5
Smp_154290.1:pep	152.75	35	7	20.7	VAL27
Smp_176160.1:pep	151.87	34	7	20.6	VAL26
Smp_154260.1:pep	151.87	34	7	20.6	VAL28
Smp_176180.1:pep	105.61	25	7	21.0	VAL9
Smp_179480.1:pep	88.87	15	4	31.2	VAL5
Smp_070250.1:pep	73.94	10	3	31.2	VAL15
Smp_002630.1:pep	21.22	4	1	26.6	VAL2
Smp_078490.1:pep	96.89 (X! Tandem)	7	1	24.6	VAL14

### Receptors and neuropeptides in *S*. *mansoni* miracidia

Approximately 43 receptors and receptor-associated proteins were identified and annotated in the proteome analysis, as shown in **S2 Table**, represented by ionotropic, G-protein coupled, kinase-related and nuclear receptors. Most of these receptors have low sequence coverage supported by LCMS spectra, which could be attributed to the limitation of protein extraction method in purifying membrane proteins. The mitochondrial import receptors (TOM34 and 40) were most abundant.

Neuropeptide and neurotransmitter receptors were also found, including an acetylcholine receptor subunit α-2 and 4 that belongs to a superfamily of ligand-gated ion channels, allowing for the flow of sodium and potassium across the plasma membrane in response to ligands such as acetylcholine and nicotine. Also an FMRFamide receptor was found by X! Tandem (**S2 Table**).

**Fig 4:**
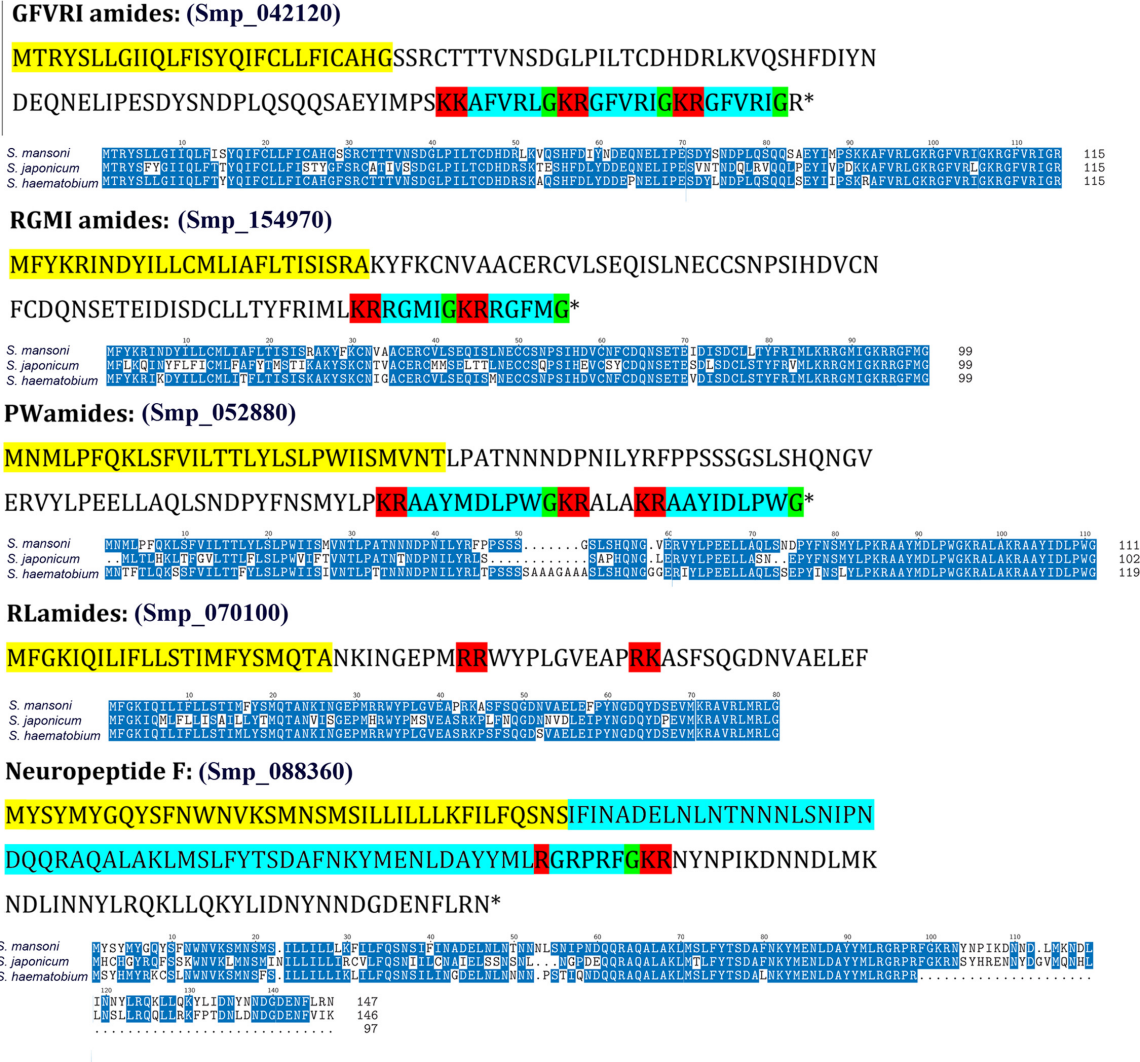
Characterisation of neuropeptides within *Schistosome mansoni* miracidia. Shown is *S*. *mansoni* annotated precursor neuropeptides, including signal peptide (yellow), cleavage sites (red), glycine for amidation (green) and bioactive peptide (light blue). Also, comparison with *S*. *japonicum* and *S*. *haematobium* precursor protein homologs. Dark blue shading indicates amino acid conservation.

No neuropeptides were identified from the proteome analysis, however, neuropeptide precursor genes were obtained from public databases for several platyhelminth species, and then used as a query to perform *in silico* BLASTp search of the *S*. *mansoni* genome, from which 5 *S*. *mansoni* neurohormone genes were identified. Translated sequences encoded RGMIamide, GFVRIamide, PWamide, RLamide and Neuropeptide F (NPF) neuropeptides (**Fig 4**and **Table 5**). Compared with *S*. *japonicum* and *S*. *haematobium* homologs, there appears to be high amino acid identity. Our RT-PCR determined that all five *S*. *mansoni* neurohormone genes were expressed at the 2h post-hatch stage. Besides the miracidia stage, GGVRI, RGMI, PWamide and NPF gene transcripts have been identified in other life-stages, including cercariae, somule and adult worms (**Table 5**and **S2 Fig**).

**Table 5:** Summary of *S*. *mansoni* neuropeptide genes identified in this study.

Name	Full length (aa)^*a*^	Signal (aa)^*b*^	Mass (kDa)^*c*^	Miracidia	Cercariae	Adult
GFVRIamide	115	28	13.2	Yes	Yes	Yes
RGMIamide	99	26	11.5	Yes	Yes	Yes
Pwamide	111	30	12.7	Yes	Yes	Yes
Rlamide	80	23	9.3	Yes	Yes	No
Neuropeptide F	147	37	17.6	Yes	Yes	Yes

^*a*^ size in amino acids of precursor neurohormone

^*b*^ predicted length of signal sequence based on SignalP

^*c*^ full-length precursor

## Discussion

In this study, to provide a comprehensive overview of the miracidia proteome, we combined the results of four search engines to assess the MS/MS spectral data. The outcome indicated distinct variations in protein identification dependent on the search engine (see **Fig 1B**), which could be attributed to differences in search algorithms and associated parameter settings [43]. This is supported by a recent benchmark study that used a pool of 20,103 synthetic peptides to evaluate peptide-spectrum matches (PSMs) using two different LCMS systems followed by the analysis with 1,800 different search engine and parameter set combinations [43]. That study found that the choice of parameter settings had a large influence on the identification performance of the search engine. Thus, we recognise that the downstream statistical analysis we used to filter PSMs could potentially generate true/false positives or negatives, as different search engines provide various fractions of correct and incorrect PSM-assignments, numbers and types of correct assignments.

Numerous *de novo* only peptides (peptide sequences that don’t have any match against the database in use) resulted from *de novo* sequenced peptide segment from the MS/MS data have been provided in this work. For example, PEAKS engine derived a total of 3,790 *de novo* only peptides with high average local confidence greater than 80% (**S1 Table**), which could be attributed to: i) the incompleteness of the protein database due to the highly repetitive *S*. *mansoni* genome, ii) short/alternative open reading frame (s/altORF) encoded peptides that were overlooked during genome annotation, and iii) contaminating mouse liver tissue attached to the isolated *S*. *mansoni* eggs that could have further attached to hatched miracidium. Herein, we provide the sequences of *de novo* only peptides as a data source for future investigation of possibilities i) and ii). Particularly for ii), the existence of s/altORF encoded peptides has been widely described in viruses [44,45,46,47] and increasingly found in humans with potential functions [48,49,50,51,52,53], including targets of anti-tumor responses in several type of cancers [50].

The enrichment analysis emphasises the importance of proteins playing roles in energy production, transportation and conversion in miracidia (**Fig 2**). It is possible that the overrepresentation of proteins in energy-related processes is due to the active development and more importantly host-seeking movement of miracidium. Thus, the signalling blockade of the key proteins on these pathways might inhibit its maturation and interfere with the snail infection. The biosynthesis of antibiotics is the KEGG pathway with large number of proteins in miracidium (70 relevant proteins with 49 enzymes), which approximately include 50% of sequences on this pathways identified from entire *S*. *manson*i proteome [1], suggesting a complex defence mechanism existing in it despite the small body size.

Amongst the 1,910 miracidium proteins identified, the most abundant proteins were housekeeping/motor proteins (tubulin, actin, myosin etc) and enzymes (reductases, transferases, proteases and peptidases) (**S2 Table**). A putative rootletin protein and ciliary outer arm dynein are present at relatively high abundance, which likely originates from the miracidia cilia. A number of putative glycoproteins with high abundance were also identified, including both secreted and non-secreted proteins; these include secretory glycoprotein k5 precursor, nuclear pore membrane glycoprotein, GPI-anchored surface glycoprotein, and so forth.

One of well represented protein identified by LC-MS/MS is the *S*. *mansoni* protein 40 (Smp-40), also known as heat shock protein-HSP20, which is in accordance with a recent report showing that this protein can contributes to 15% of the soluble proteins of miracidia [13]. Smp-40 is one of a number of previously identified soluble egg antigens (SEA) and soluble worm antigen proteins (SWAP) [54], that has been investigated for its role in blastogenic reactions and granuloma responses, and as serodiagnostic target. As a member of the small heat shock protein (sHSP) family, it helps to capture unfolded proteins that form stable complexes [55], resulting in efficient disaggregation of the protein aggregates. The amphitropic sHSPs have been shown to associate with membranes, despite the lack of transmembrane domains or signal sequences. Proteins homologous to Smp-40 have previously been identified in *S*. *japonicum* [56], as well as the tapeworms *Taenia saginata* [57] and *Echinococcus multilocularis* [58], and the lung fluke *Paragonimus westermani* [59]. In addition to HSP20, HSP16, 27 and 40 were also identified in this study. A set of sHSPs are highly upregulated in the ‘dauer’ larvae of *Caenorhabditis elegans* [60], which is a stage induced by unfavourable growth conditions. The dauer state is resistant to environmental stresses by significantly reducing metabolic activity, yet a functional chemosensory system and rapid responsive movement can be maintained [61,62,63]. Miracidia face similar physiological stresses after hatching and during host-finding; thus, the abundance of sHSP could offer the miracidium protection against stress. Moreover, it has been suggested that the sHSPs work in an ATP-independent manner [64,65], ensuring that glucose reserves are not overused.

A 78 kda glucose-regulated protein (GRP78) was also identified with high abundance, which is a member of the heat shock protein 70 (HSP70) multigene family (**Table 3**). This finding extends upon a previously reported study which found that chaperones are the most abundant proteins in the *S*. *mansoni* egg [13]. The expression of HSP70 has been noticed throughout the development of egg, which suggests its multi-functional roles, including protein maturation in the reticulum of embryogenic processes [66] and in mitochondrial biogenesis [67]. GRP78 is involved in the folding of nascent proteins within the endoplasmic reticulum and the translocation of secretory proteins [68], thus it could play a similar role in stabilising macromolecular structure for miracidium proteins destined for secretion. The GRP78 itself has also been found to be released from cells and into the peripheral circulation [69]. We also identified the predicted secreted protein alkaline phosphatase in the miracidia proteome (see **Table 3**). Alkaline phosphatase in *S*. *mansoni* sporocysts has been speculated to function in nutrient uptake and might serve as a marker of infection of *B*. *glabrata* snails [70].

Two mucin-like proteins were identified, with N-terminal domains containing a variable number of tandem repeats. Polymorphic Mucins (*Sm*PoMucs) act as key strain markers since they vary between the strains of *S*. *mansoni* known to infect *B*. *glabrata* [71]. Their proposed function is to interact with the host snails Fibrinogen Related Proteins (FREPs) [72], and other proteins associated with the secondary immune response of the snail [73,74]. Of note, it has been observed that the *Sm*PoMuc genes are only expressed in stages that interact with the host snail [17] and that each individual miracidium shows significant sequence differences [75].

The secreted glycoprotein kappa-5 (k5) was first characterised as an immunogenic egg glycoprotein from *S*. *mansoni*, and is probably a key mediator of host-parasite interactions since it triggers a pronounced IgE response in the human host [76]. Similarly, its role as an authentic granuloma-inducing component in snail hosts is worthy of study, and to compare with its similar function in the mammalian host [77]. The secreted calreticulin-like protein is a Ca^2+^ binding/storage protein, which is highly conserved, being found in a number of different animal taxa. It has recently been considered as a novel antigen for the detection of anti-*S*. *mansoni* antibodies as it possesses the advantage of cheaper and easier production compared with the soluble egg antigen that is traditionally used [78].

Previously, four VAL protein members have been found by the transcriptomic analysis of different life stages of *S*. *mansoni* [37], and later a further 24 members were discovered [79]. Although the biological function of VAL proteins still remains elusive, it has been proposed that may play an important role in the host-parasite interaction [80]. Gene expression of some VAL proteins (VAL6 and 7) is restricted to the oral and ventral suckers of adult worms and in the oesophageal gland [81], while VAL 2, 3, 5 and 9 have inflammation-inducing properties [16]. The VAL 9 protein has additionally been found in soluble egg products and miracidia/sporocysts, and is potentially involved in tissue reorganisation/extracellular matrix remodelling [82].

We found a GPI-anchored surface glycoprotein within the miracidia proteome, however, its role in miracidia remains unclear. The GPI-anchored surface glycoproteins are also found in adult *S*. *mansoni*, localised to the outer surface [83,84]. A saposin protein was identified, displaying the characteristic six conservative cysteines that form three disulphide bridges, previously reported within the gastrodermis of *S*. *mansoni* adult and cercariae [85]. Saposins exist widely in most animals; their function varies but seems to relate to interactions with lipids. In *Clonorchis sinensis*, it has been shown to lyse ingested host cells for nutritional purposes [86], while in the case of *Fasciola hepatica*, it works externally to the parasite as a secreted product [87], thus implicating it in a feeding role.

The interleukin-4-inducing protein precursor was first identified with high expression in the eggs of *S*. *mansoni*, where it can stimulate the mammalian host to rapidly release large amounts of interleukin-4 (IL-4) [88]. IL-4 is a key promoter of a Th2 response, via an IgE-dependent but non-antigen-specific mechanism. We found that this protein is present in abundance in miracidia (**Table 3**), which contrasts a report that this protein is produced exclusively by the eggs [89]. As a secreted glycoprotein, it could play a similar role in the body of the intermediate snail host.

Cathepsins D and L were also identified in our miracidia proteome. It has been suggested, from an evolutionary perspective, that cathepsins D and L are two key proteases (others include cathepsins B, C and legumain) that allow for efficient digestion in early metazoans. In addition, studies of parasitic helminths have suggested that these proteases function as digestive enzymes in vertebrates earlier than the evolution of the pancreas and the serine [90]. The aspartic protease cathepsin D has been found in the schistosome gut as an apical enzyme in digesting hemoglobin released from ingested erythrocytes [91]. The reduction of cathepsin D transcripts leads to significant development retardation *in vitro*, aspartic protease enzyme activity suppression and the malfunction of the digestion of host hemoglobins in the gut of schistosome adults [92]. Vaccination of sheep and cattle with purified *F*. *hepatica* cathepsin L elicits protection (50–73%) and anti-fecundity effects against challenge infection [93]. Thus, these two proteases might play certain roles in miracidia metabolism, for example, at post-infection they may regulate the digestion of host snail’s haemoglobin [94].

The identification of abundant mitochondrial import receptors may correlate with the high energy requirement for miracidium growth, development and extensive host-seeking activity within the first 5–6 h post-hatch. TOM34 was found to act as an important component of the translocation machinery of the outer mitochondrial membrane [95], while TOM40 forms hydrophilic channel for the transport of preproteins [96]. The presence of epidermal growth factor, netrin and ribosome receptors in miracidia additionally supports the requirement for active growth. Human low-density lipoprotein (LDL) binds to the surface of schistosomula adult and cercariae via LDL receptor [97]; the identification of LDL receptor in miracidium indicates a similar scenario might take place between miracidium and host snail.

Neuropeptides are used by neurons to communicate with cells. Despite little understanding of their role in schistosome miracidia, we expected that they would be important as key endogenous mediators during host-seeking behaviours, required for locomotion, sensory responses and basic catabolism of endogenous glycogen stores. In this study, although no neuropeptides were identified from the miracidia proteome analysis, 5 neurohormone genes were identified by RT-PCR. Of the 5 neurohormones, NPF has been the most extensively studied across the phylum Platyhelminthes. The neuropeptide Y superfamily has important functions in both vertebrate and invertebrate taxa with two distinguishable types of peptides, neuropeptide F-like (NPF), most notably found in invertebrates, and NPY-like peptides found typically in vertebrate species [98]. However, analysis of recent planarian genomes has shown that a specific family of NPY (specifically NPY-1,4,9) genes could encode for both NPY-like and NPF-like neuropeptides, suggesting an evolutionary relationship between vertebrate NPY and invertebrate NPF peptides [98]. Neuropeptide F within invertebrates has been linked to feeding, various locomotion roles, reproduction and stress responses [99]. In adult worms of *S*. *mansoni* NPF functions in modulation of sensory systems, motor function, reproduction, and feeding [100]. However, as the two latter functions can be excluded from miracidium, with neither the need to feed, nor reproduce, it can be predicted to play roles in locomotion and in varying sensory modulation [100].

A report has indicated possible inter-species differences in the functions of GFVRIamide, which fall into the broad I/Lamide family, due to the apparent lack of conservation and difference in expression [101]. Recently described, the novel GFVRIamide has been linked to potential neuromuscular and interneuronal communication roles in the adult worm, suggesting a putative role also in the miracidium [101]. RLamide and RGMIamide are newly described novel peptides that fall into the same I/Lamide family, and along with the PWamides all have unknown phenotypical expression across the phylum Platyhelminthes. A high level of inter-species conservation, which was seen within the multi sequence alignment, suggests potential putative roles for these neuropeptides, with neuropeptides such as NPF, having similar and consistent functions across invertebrate species. However, obtaining a next-generation transcriptome is recommended so as to identify further novel neuropeptides and associated receptors, where functions of expressed and novel neuropeptides can be determined using gene knockout techniques. Species conserved neuropeptide receptor systems make for potential novel drug targets, and further exploration of the function of these receptors systems at the miracidial level is recommended, with future studies to elicit the expression of each neuropeptide throughout the miracidium life-span to analyse neuropeptide expression patterns.

## Conclusions

Our study has revealed the proteome of the *S*. *mansoni* miracidium, uncovering important molecules in biological pathways that play key roles, such as in glutathione metabolism, phenylalanine metabolism, phenylpropanoid biosynthesis and the TCA cycle. The biological processes related to energy regulation are highly enriched at this stage, with a large ratio of proteins having functions of ATP/GTP relevance; in addition, the enrichment of protein folding/polymerisation and microtubule-related processes further indicates the active development of miracidia. It has highlighted the principal secreted proteins the miracidium releases, some of which are possibly involved in environment adaption, host seeking and invasion, including sHSP, HSP70, mucins, VALs, interleukin 4, GPI-anchored glycoproteins and others; in addition, identified receptors also suggest that active biological processes occur in miracidia, including immune response, phosphorylation and epithelial cell growth. The neuropeptides, including RGMIamide, GFVRIamide, PWamide, RLamide and NPF, were identified through gene expression analysis. These are highly conserved between schistosome species.

## Supporting Information

S1 Fig The distribution of all KEGG pathways identified from miracidia proteins. (TIF)

S2 Fig Graphs show relative mRNA concentration of neuropeptide at cercariae (C), somulae (3S and 24S) and adult schistosome (A) stages. (TIF)

S1 Table Identification of *Schistosome mansoni* miracidia proteins by PEAKS, MS Amanda, X! Tandem and OMSSA, including summary of proteins, supporting peptides and *de novo* only peptides identified by LC-MS/MS, see Methods for more details. (XLSX)

S2 Table Summary of annotation of identified proteins, predicted secretory proteins and receptors. (XLSX)

S3 Table Summary of gene ontology analysis at all levels, Fisher’s exact test result and KEGG analysis. (XLSX)
