# Supplementary figures and images for: Proteomic Analysis of the Schistosoma mansoni Miracidium
Source: PLoS One. 2016 Jan 22;11(1):e0147247. doi: 10.1371/journal.pone.0147247 (PMC4723143; doi:10.1371/journal.pone.0147247)

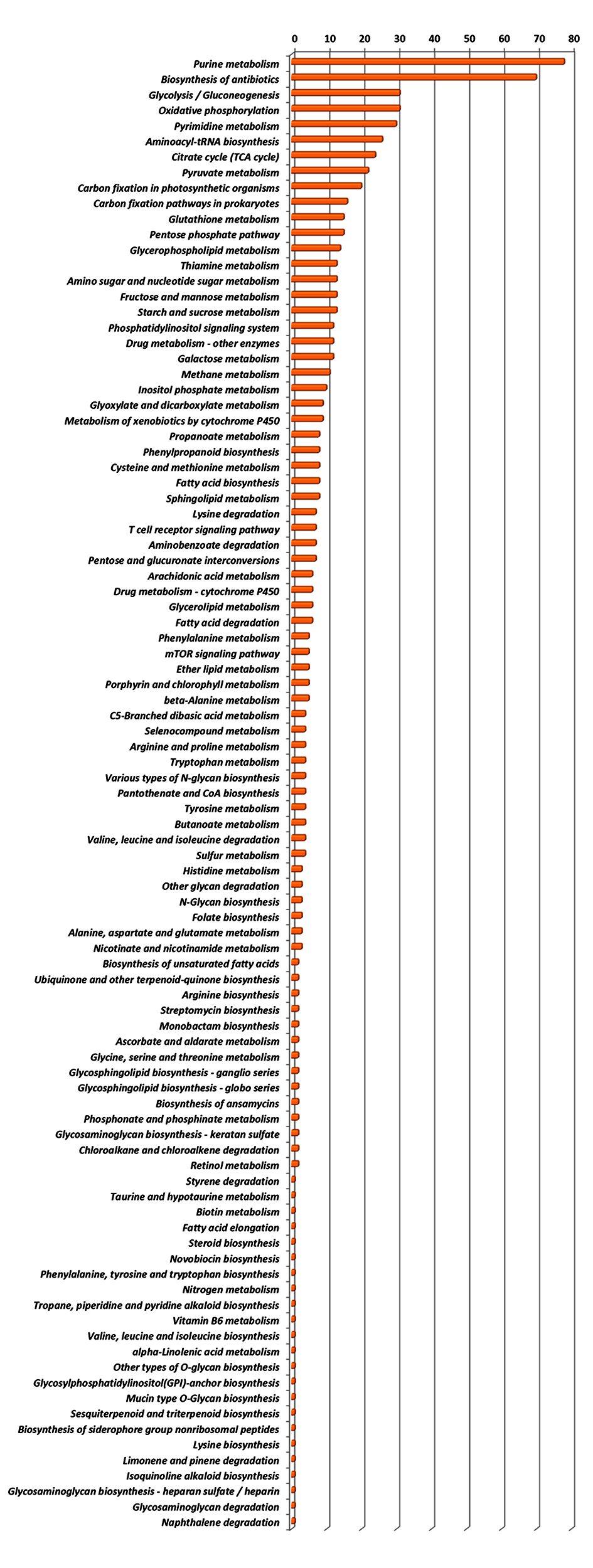

Supplement: S1 Fig — (TIF) [file pone.0147247.s001.tif]

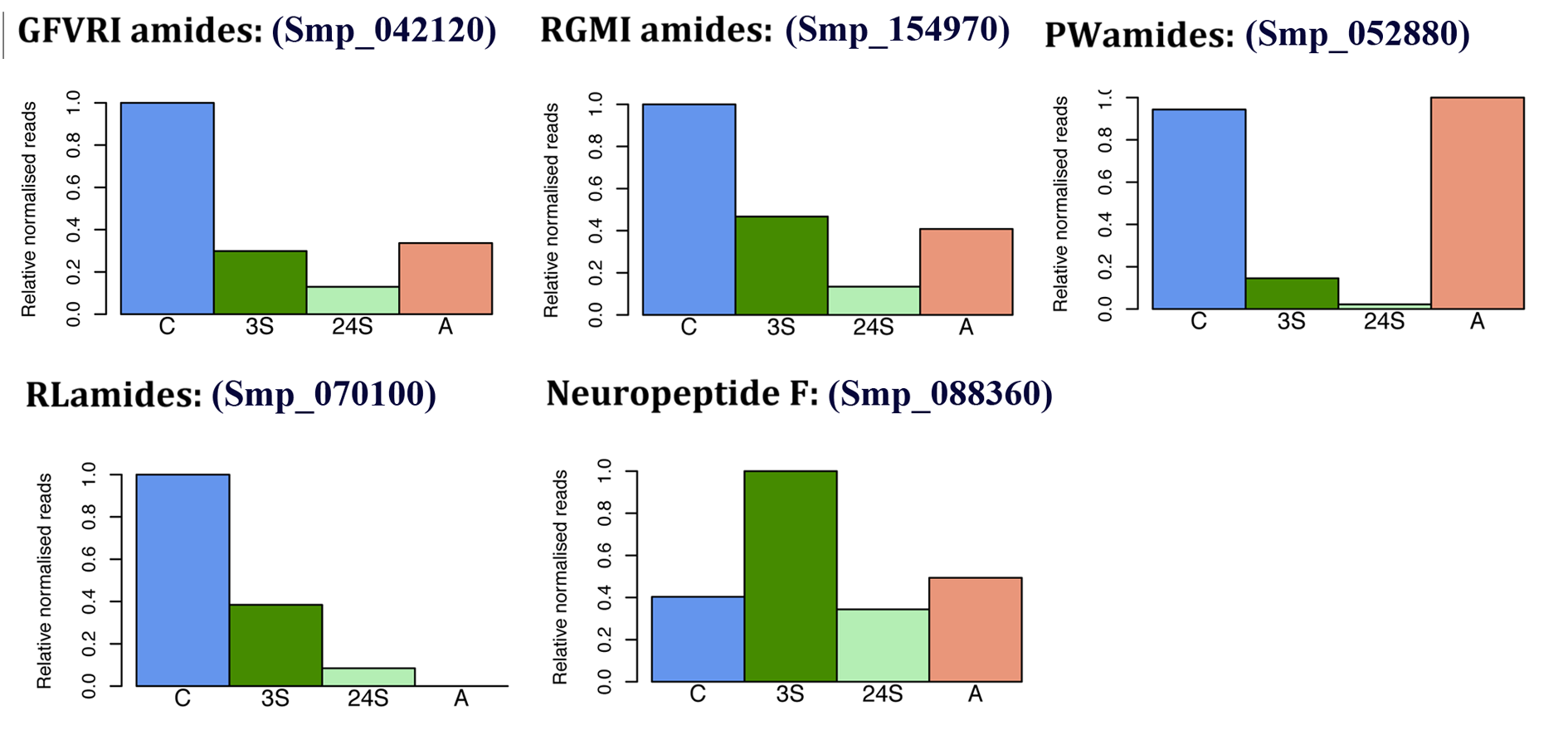

Supplement: S2 Fig — (TIF) [file pone.0147247.s002.tif]
